# Individual Utilities of Life Satisfaction Reveal Inequality Aversion Unrelated to Political Alignment

**DOI:** 10.1007/s11205-026-03854-4

**Published:** 2026-05-13

**Authors:** Crispin Cooper, Ana Fredrich, Tommaso Reggiani, Wouter Poortinga

**Affiliations:** 1 Department of Computer Science and Informatics, Cardiff University, Cardiff, UK; 2 Cardiff Business School, Tommaso Reggiani, Cardiff University, Cardiff, UK; 3 School of Psychology, Cardiff University, Cardiff, UK; 4 MUEEL Lab, Masaryk University, Brno, Czechia; 5 Welsh School of Architecture, Cardiff University, Cardiff, UK

## Abstract

How should well-being be prioritised in society, and what trade-offs are people willing to make between fairness and personal well-being? We investigate these questions using a stated preference experiment with a nationally quasi-representative UK sample (*n* = 300), in which participants evaluated life satisfaction outcomes for both themselves and others under conditions of uncertainty. Individual-level utility functions were estimated using an Expected Utility Maximisation (EUM) framework and tested for sensitivity to the overweighting of small probabilities, as characterised by Cumulative Prospect Theory (CPT). A majority of participants displayed concave (risk-averse) utility curves and showed stronger aversion to inequality in societal life satisfaction outcomes than to personal risk. These preferences were unrelated to political alignment, suggesting a shared normative stance on fairness in well-being that cuts across ideological boundaries. The results challenge use of average life satisfaction as a policy metric and support the development of nonlinear utility-based alternatives that more accurately reflect collective human values. Implications for public policy and well-being measurement are discussed.

**Supplementary Information:**

The online version contains supplementary material available at 10.1007/s11205-026-03854-4.

## Introduction

Life satisfaction has gained substantial traction as a metric for public policy evaluation, with much current interest in its use as a direct measure of outcomes in place of traditional economic indicators such as income or Gross Domestic Product (Carney, 2020; Cato, 2008; Frijters & Krekel, 2021; Layard, 2006). Advocates of the approach argue that life satisfaction captures a broader, more subjective conception of welfare that aligns more closely with what individuals value. However, life satisfaction-based policymaking faces a core conceptual challenge: life satisfaction is not fungible in the way monetary metrics are. A change from, e.g., 6 to 7 on a self-reported satisfaction scale may not reflect the same change in utility across individuals, and the scale itself lacks a natural unit that permits aggregation or comparison in cardinal terms.

Despite this, much empirical and policy-focused work treats life satisfaction scores as if they are directly comparable and additive across individuals. Researchers routinely average life satisfaction as though each unit represents a constant and interpersonally comparable increment of welfare. This practice is rarely justified and often implicit, yet it underpins a substantial body of work in well-being economics and public policy (Bronsteen et al., 2013; Cooper, 2020).

A further issue with averaging well-being scores is that the practice implicitly endorses a Benthamite utilitarian conception of social value, in which welfare is identified with the total amount of ‘good’. In contrast, a Rawlsian conception holds that social value depends primarily on the well-being of the least well off (Fleurbaey, 2011; Layard, 2016). These are only two of many possible positions on a broader spectrum of possible social welfare functions. In policy evaluation, it is inevitable that some position along this spectrum is adopted, whether implicitly or explicitly; yet this choice is rarely acknowledged in applications of life satisfaction data.

One proposed approach to choosing such a position is to consult the public on the values they prioritize (Frijters et al., 2020), a research direction also identified as a high priority by Layard and De Neve (2023). The contribution of this paper is to address this gap empirically by eliciting public preferences over trade-offs between distributions of life satisfaction under uncertainty. The aim is to estimate the utility functions individuals apply to life satisfaction when evaluating both personal risk and societal inequality, and demonstrate the potential policy relevance of these functions. We do this through a stated preference experiment in which participants choose between different life satisfaction distributions, varying in both inequality and average well-being, under a “veil of uncertainty” regarding their own position within the distribution.

The specific objectives are,

1. to validate comprehension and reliability of responses, ensuring that observed preferences reflect meaningful and interpretable trade-offs.
2. to estimate personal and social utilities of life satisfaction, and how they vary by political alignment,
3. to demonstrate the application of these utilities in an exploratory aggregation of UK life satisfaction data,
4. to test sensitivity of results to application of small probability weighting.

The veil of uncertainty (or veil of ignorance) has a long-standing tradition in welfare economics and theories of distributive justice (Rawls, 1971). Its central premise is that one society may be considered more just than another - even if both are unequal - if individuals would rationally choose to live in it while unaware of their eventual position within the distribution. This philosophical framework thus posits a connection between individuals’ tolerance for personal risk and their acceptance of societal inequality, a link that we explicitly test in the context of life satisfaction.

By eliciting decisions made under uncertainty about individuals’ own positions, we infer implicit preferences regarding inequality and risk in terms of life satisfaction. We estimate these preferences using flexible, nonparametric models fitted to experimental standard gamble data. This approach allows us to move beyond the assumption of additive life satisfaction and instead develop a framework that respects the ordinal and context-dependent nature of life satisfaction measures.

The remainder of the paper is structured as follows: Section 2 reviews relevant background literature, including decision-making theories, attempts to compute utilities for the subjective life satisfaction scale, and the link between political alignment and views on inequality. Section 3 explains the survey design and analytical approach used. Section 4 presents the results, including response validation, utility estimation, application to UK life satisfaction data, and sensitivity analysis. Sections 5–7 discuss the implications, limitations, and conclusions of the research.

## Literature Review

### Utilities of Life Satisfaction, Expected Utility Maximization and Prospect Theory

Among the various measures of subjective well-being, this study focuses on *life satisfaction*, which is the most widely used and institutionally accepted indicator and is closely aligned with evaluative life outcomes (Fujiwara & Campbell, 2011). Life satisfaction is typically operationalised using a single-item question of the form: “On a scale of 0 to 10, where 0 is ‘not at all’ and 10 is ‘completely’ […] Overall, how satisfied are you with your life nowadays?” (Office for National Statistics, 2018). Its widespread use in official statistics and policy evaluation makes it a natural focal point for examining aggregation and valuation issues.

A parallel development can be seen in the health domain, where Quality Adjusted Life Years (QALYs) are used to evaluate health outcomes (Brazier et al., 2016; Frijters et al., 2020). Recent work proposes the development of a comparable measure not just for health, but overall life satisfaction, termed the Well-Being Adjusted Life Year (WELLBY), which weights each year of life by a person’s life satisfaction score (Frijters et al., 2024). The WELLBY applies a linear weighting, thus implicitly taking a Benthamite position on social value.

At the core of life satisfaction valuation lies the concept of utility, commonly defined as the quantity individuals seek to maximise in decision-making. Utilities are typically inferred from observed choices in experimental settings, under the assumption that individuals are more likely to choose the option they perceive to yield higher utility. The exact interpretation of utility, however, depends on the underlying theoretical framework. Expected Utility Maximization (EUM), developed by von Neumann and Morgenstern (1953), is a normative theory specifying how rational agents should make decisions under uncertainty. In contrast, Cumulative Prospect Theory (CPT) (Kahneman & Tversky, 1979; Tversky & Kahneman, 1992) provides a primarily descriptive account of observed decision-making biased by loss aversion and probability weighting.

The choice of which framework to use (EUM vs. CPT) is a matter of ongoing debate. From an empirical perspective, and unsurprisingly given its aims, CPT has provided a closer overall fit to observed human decision-making (Barberis, 2013; Gonzalez & Wu, 1999). A meta-analysis of 106 transportation studies found CPT’s reference-dependent framing and diminishing sensitivity consistently outperformed EUM (van de Kaa, 2010). Overall differences in performance, however, mask differences in both participants and context. Bruhin et al. (2009) finds heterogenous decision making behaviours among participants, with 80% following CPT and 20% EUM, and hence recommends to use a mix of preference theories. In a health context, Abellan-Perpiñan et al. (2009) find that CPT better predicts decision under risk, but not intertemporal decisions.

In a policymaking context, there are moral considerations beyond the empirical fit of the chosen framework. Beyond its empirical origins, CPT has also been proposed as a normative framework, used to ‘de-bias’ observed choices in order to infer latent utilities (Bleichrodt, 2002). But this is not uncontroversial: inferring preferences that differ systematically from stated choices raises questions about democratic legitimacy, particularly when policy decisions are justified on the basis of public values. Although representative democracies allow some scope for interpretation of public preference (Sumption, 2019), it would nonetheless be a bold claim to assert that one’s own statistical model of voter preferences is more accurate than what voters themselves report. Barberis (2013), reviewing 30 years of CPT, concludes that “we do not, as yet, have a full understanding of whether loss aversion or probability weighting should be thought of as mistakes”.

In practice, policymakers have tended to rely on EUM rather than CPT, particularly in health-related decision-making (European Medicines Agency (EMEA), 2010; National Institute for Clinical Excellence (NICE), 2013; Reed Johnson et al., 2013); a characterisation that is also acknowledged by critics of the approach (Abellan-Perpiñan et al., 2009; Attema et al., 2013). Importantly, both EUM and CPT converge on a common formal concept of loss aversion: the relative weighting of losses compared to equivalent gains. Under EUM, loss aversion is directly reflected in the curvature of the utility function, whereas CPT separately encodes loss aversion in valuation functions applied to the ‘true’ utility change. CPT also corrects for probability weighting.

Given the above considerations, the current study adopts a theory-agnostic position based on relative valuation of loss and gain. We achieve this by computing EUM-style curves without correcting for loss aversion, and then measuring risk and inequality aversion from their curvature: the reader is free to interpret such results either as ‘true’ risk/inequality aversion or as irrational bias, depending on their theoretical leanings. We do, however, examine the sensitivity of results to the inclusion of CPT-style probability weighting functions, to test the effect of biases related to small probability losses, when computing the utility curves.

If loss aversion is present in decision-making, then under EUM the utility function must be nonlinear. This challenges several arguments for utility linearity with respect to life satisfaction, as summarised by Frijters and Krekel (2021). First, it is argued that asking participants to rate life satisfaction on a linear scale encourages them to treat changes of ± 1 as equivalent across the scale - such that a move from 8 to 9 is perceived as comparable to a move from 3 to 4. Second, it is found that prediction errors in life satisfaction are homoscedastic, implying that the likelihood of over- or underestimation is constant across the scale. Third, it is claimed that individuals can estimate the life satisfaction scores of others with reasonable accuracy.

We argue that these points conflate perceptual linearity of life satisfaction with utility linearity. Even if a life satisfaction scale is perceptually linear, it does not follow that utility is a linear function of the life satisfaction scale. If choices consistent with loss aversion are found, then under EUM the utility function is by definition nonlinear. Under CPT such choices could reflect nonlinear utilities, nonlinear utility valuation, biased probabilities, or a combination of all three.

Two notable experimental studies provide an empirical precedent to challenge the assumption of linearity. Peasgood et al. (2018) used a Time Trade-Off (TTO) method to elicit utility weights for life satisfaction, finding a mildly concave (risk-averse) utility curve. This method avoids bias from probability weighting. Mukuria et al. (2023) reported a more pronounced concave utility curve on the EQ-HWB-S health and well-being scale; although not explicitly discussed, this is visible in the fact that lower states on the perceptually linear EQ-HWB-S scale were found to have greater utility decrements (until a utility of zero was reached, beyond which, states were rated worse than dead and show the opposite). This is based on TTO, augmented by a discrete choice experiment requiring trade-off between different components of the multidimensional scale.

While these studies complement the present work, there are several important differences. Peasgood et al. (2018) did not attempt to anchor life satisfaction scale positions using interpersonally comparable vignettes, while Mukuria et al. (2023) focused on the EQ-HWB-S rather than the life satisfaction scale. Moreover, neither study directly presented participants with choices involving trade-offs between risk and inequality, which are central to policy evaluation and social welfare aggregation. Finally, neither examined how such preferences relate to political ideology. The present study addresses these gaps by eliciting preferences over life satisfaction distributions under uncertainty, allowing for the joint estimation of risk and inequality aversion.

### Relationship Between Political Alignment and Inequality Aversion

Part of the contribution of the present study is to measure individual aversion to well-being inequality, and test its relationship with political alignment. This section reviews the literature outlining why we might expect these factors to be linked.

A large interdisciplinary literature documents a systematic association between political alignment and tolerance of inequality. Individuals on the political left tend to express lower tolerance for unequal outcomes and stronger support for redistribution, whereas right-leaning individuals are more accepting of inequality, often emphasizing efficiency, incentives, and market-based fairness. Canonical political economy models formalize this relationship by linking income position and voting behaviour to redistribution (Meltzer & Richard, 1981). Empirical evidence confirms that ideology remains a central predictor of redistributive preferences across advanced democracies (Alesina & Giuliano, 2011).

More recent work refines this account by identifying the belief-based mechanisms through which ideology operates. Inequality tolerance is shaped less by material self-interest alone than by beliefs about fairness, responsibility, and opportunity. Experimental evidence shows that individuals are more willing to accept inequality when disparities are attributed to effort rather than luck (Cappelen et al., 2010). Similarly, beliefs about intergenerational mobility strongly influence redistribution preferences and vary systematically with political alignment (Alesina et al., 2018). Stantcheva (2024) further argues that policy preferences reflect broader perceptions and narratives about the origins and consequences of inequality. In this context, ideological disagreement often concerns causal interpretation rather than inequality levels per se.

A parallel behavioural literature highlights the contextual sensitivity of inequality preferences. Support for redistribution responds strongly to framing and information provision (Kuziemko et al., 2015). Fisman et al. (2017) provide evidence that individuals’ distributional preferences cannot be reduced to a single left-vs-right dimension, as people simultaneously express distinct concerns for efficiency, equality, merit, and social welfare that do not always align in a consistent ideological ordering. Extending this perspective, Fazio and Reggiani (2023) show that institutional reference points and contextual comparisons shape both perceptions of inequality and political preferences. Ideological differences therefore depend on how inequality is benchmarked and evaluated relative to salient reference standards.

Political psychology complements these findings by emphasizing system-level motives. System Justification Theory (Jost et al., 2018) posits that individuals are motivated to defend and rationalize existing social arrangements, including unequal ones. Although this tendency is more strongly associated with conservative orientation, system-justifying motives are not confined to the right and coexist with egalitarian impulses across the ideological spectrum. This generates heterogeneity within political camps and attenuates simple ideological predictions.

Taken together, the evidence establishes a robust association between political alignment and tolerance of inequality, particularly in income-related domains. At the same time, this relationship appears to be conditional. Inequality tolerance is mediated by beliefs about fairness and mobility, shaped by cognitive framing and reference points, and influenced by psychological motivations to justify or challenge the status quo. Ideological differences are therefore neither fixed nor uniform across contexts. Understanding these contingencies is essential for interpreting the link between political alignment and domain-specific distributive preferences.

## Methods

### Sample and Recruitment

The sample consisted of 300 participants, using stratified quota sampling to reflect the UK population in terms of age, sex, and political affiliation. While quota sampling does not constitute probability sampling and may be subject to selection biases that quotas cannot fully eliminate, this approach was chosen to ensure broad demographic coverage across these groups. Age and sex were included due to their known associations with risk-taking behaviour (Bonem et al., 2015; Byrnes et al., 1999). Political affiliation was included because the study concerns distributive preferences and attitudes toward social inequality, which are likely to be shaped by ideological orientation.

Sample size requirements for the discrete choice experiment were estimated, using established heuristics. Orme’s rule of thumb suggested a minimum of 125 participants, while Assele’s more recent method indicated a threshold of 75 (Assele et al., 2023). The final sample exceeded both recommendations to increase representativeness. Sample characteristics are shown in Table 1.

**Table 1:** Characteristics of the sample at time of recruitment

Sex	Female					Male					Female	Male	All
Age	18–24	25–34	35–44	45–54	55+	18–24	25–34	35–44	45–54	55+	All ages		
Conservative	1	4	4	4	16	1	3	3	4	14	29	25	54
Green party	2	2	2	2	3	2	2	2	2	3	11	11	22
Labour	11	14	14	12	22	11	13	13	12	19	73	68	141
Liberal democrats	1	3	3	2	5	1	2	2	2	5	14	12	26
Reform UK	1	2	2	3	12	1	1	1	3	11	20	17	37
SNP	1	1	1	1	1	1	1	1	1	1	5	5	10
Other	1	1	1	1	1	1	1	1	1	1	5	5	10
All	18	27	27	25	60	18	23	23	25	54	157	143	300

Participants were recruited through Prolific (www.prolific.com) and completed the survey on 9 April 2025. As with other online platforms, samples recruited via Prolific should be considered quasi-representative rather than fully representative of the general population, although a growing literature shows that non-probabilistic samples can be sufficient within limitations (Gelman et al., 2016; Rivera, 2019). Prolific has been found to yield high-quality and diverse samples in recent comparative works (Douglas et al., 2023). Prior to the main study, the survey was piloted with 20 participants under live video call supervision. This pilot served two purposes: to test the survey instrument and to provide a benchmark against which the quality of unsupervised responses in the main study could be assessed.

Standard data quality checks were included, in line with best practice in online survey research (Casey et al., 2017; Chmielewski & Kucker, 2020; Lovett et al., 2018; Pyo & Maxfield, 2021; Robinson et al., 2019). These included simple attention checks (e.g. “I am currently completing an online survey”), as well as monitoring of completion times to flag unusually fast responses that might indicate disengagement.

Political orientation is a complex construct that can vary across contexts and is usually conceived as multidimensional (Feldman & Johnston, 2014). From a policymaking perspective, however, this study examines whether those who vote for different parties within the UK can nevertheless agree on the desired outcomes in the distribution of life satisfaction. For this reason, political orientation is firstly measured by asking party affiliation - how participants would vote (if they were able to) in an election tomorrow. Secondly, as left/right orientation is typically associated with tolerance of inequalities (see Sect. 2.2), we measure left/right alignment as a single variable to permit correlational analysis. Given the context-dependent nature of political alignment, and constraints on overall survey length, this is operationalized through five items capturing economic and social attitudes from the British Social Attitudes Survey, which is long established and developed within the UK context (NatCen Social Research, 2017). Responses to these questions were summed to form a single political alignment variable for analysis. The survey questions are provided in the Supplemental Material.

### Vignettes

To ensure comparability in how participants interpret and use the life satisfaction scale, we employed a vignette-based approach (Angelini et al., 2014). Participants were asked to rate the life satisfaction of fictitious characters in a series of hypothetical life situations. These ratings provide insight into how each respondent applies the 0–10 life satisfaction scale, allowing us to control for individual differences in scale use.

Two distinct conditions were used:

1. **Gambles-first condition**: Participants were first asked to make gambles (see following section) between a set of life situations (i.e., the vignettes), and then subsequently rate the life satisfaction of each situation. This approach maximises separation between the decision task and the respondent’s interpretation of the life satisfaction scale, thereby reducing contamination between scale use and utility elicitation.
2. **Life satisfaction-first condition**: Participants were first asked to rate the life satisfaction of each situation and then make the gambles between different life situations (i.e., the vignettes) using these ratings as reference points.

We used these different conditions to control for biases that may be unique to each approach. The life satisfaction-first condition could inadvertently encourage those with prior knowledge of probability and expected value to compute expected outcomes using an EUM framework. But by simplifying life situations to positions on a life satisfaction scale we also potentially reduce cognitive load, which is known to be crucial to maintain response efficiency in choice modelling in particular (Reed Johnson et al., 2013). Given that both approaches have distinct methodological advantages, we randomly assigned participants to one of the two conditions and later tested for systematic differences in responses across the two groups.

**Fig. 1:**
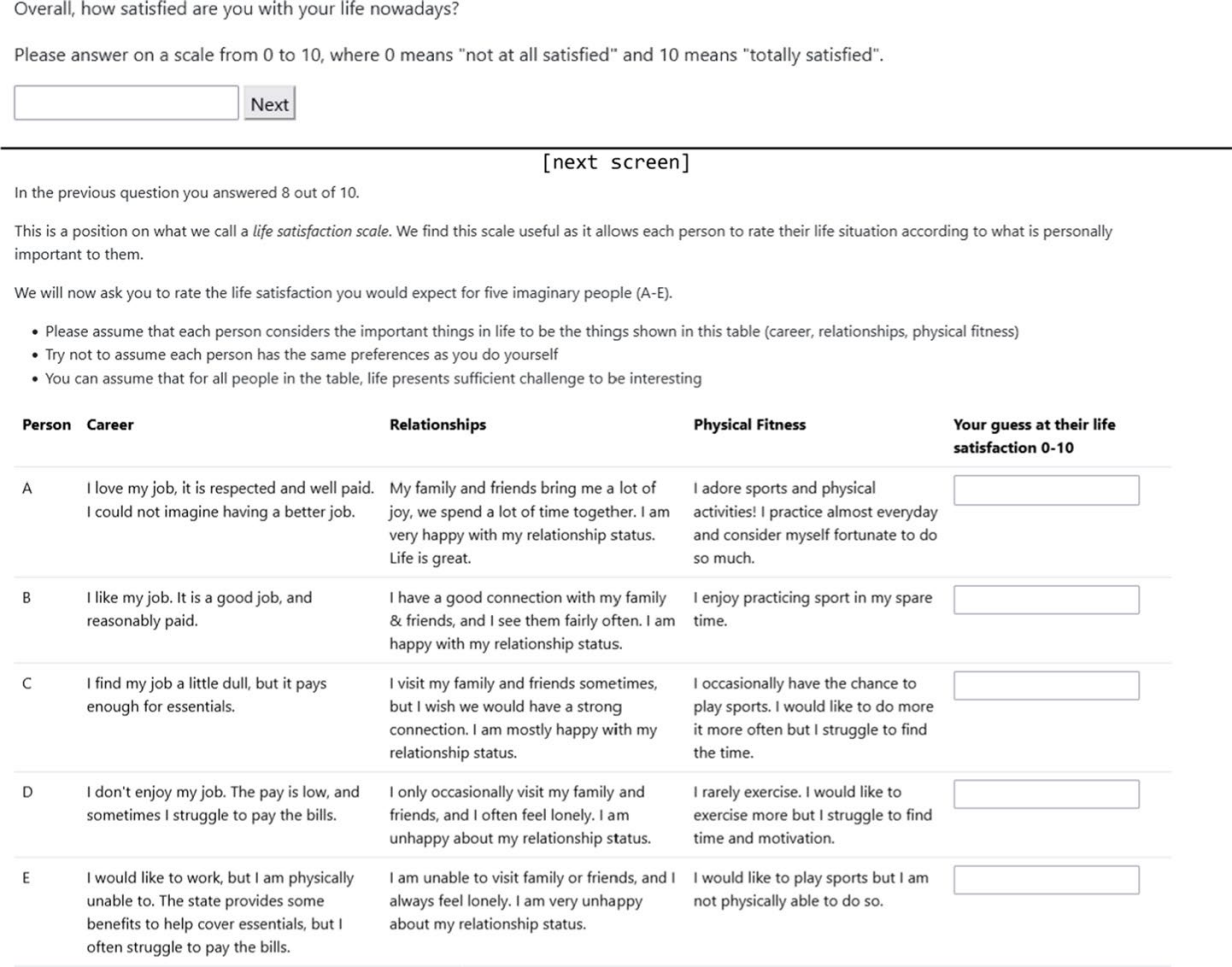
Life situations (vignettes) used for the survey

The vignettes (see Fig. 1) served three purposes: first, to familiarise participants with the life satisfaction scale; second, to provide guidance on how the hypothetical scenarios should be interpreted, emphasising that participants may hold different values from those of the vignette character, but should nonetheless evaluate life satisfaction from the character’s perspective rather than their own; and third, to establish a common reference point for gamble decisions. Each vignette described the character’s career, relationships, and physical fitness. Vignettes A–C reflected generally healthy life states with varying levels of satisfaction. Since the differences among them do not stem from health conditions, all three could reasonably be assigned a weight of 1.0 within the QALY framework. In contrast, vignettes D and E described increasing struggles with mental health, with E also involving major physical health issues, implying that both would be evaluated with a lower QALY weight.

If participants provided ratings that violated logical orderings, for example, by rating a clearly better scenario lower than a worse one, they were given the opportunity to either revise their responses or to provide an explanation.

### Gambles

To elicit individual utility functions, we employed a series of standard gamble tasks, structured around two core decision contexts:

1. **Personal risk decision (medical context)**: Participants were asked to choose between a guaranteed life situation and a uncertain alternative with known probabilities of resulting in either a better or worse life outcome, or in some cases, death. The medical context was chosen for personal risk decisions, to facilitate participants to imagine a one-time, irreversible decision. While framing questions within this domain may shape participant’s responses, this was deemed preferable to using more abstract hypothetical framings, which are known to decrease respondent engagement and efficiency (Reed Johnson et al., 2013). Depending on the survey condition, options were either labelled (A-E) or defined by life satisfaction scores derived from previous vignette ratings.
2. **Societal inequality decision (policy-maker context)**: Participants were asked to adopt the perspective of a policymaker choosing between two distributions of outcomes across a population. One policy offered equal life satisfaction outcomes for all, while the other produced unequal outcomes - benefiting some while disadvantaging others, potentially including death. To reinforce impartiality, participants were reminded that they themselves would be affected by the policy but would not know in advance whether they would benefit or lose out: “The policy will affect you as well, though you don’t yet know whether you will benefit or be negatively affected.”

Each participant completed the following three blocks of gambles, presented in randomised order within each block:

1. **Four personal risk gambles** between triples of adjacent life states on our ordinal scale, to estimate utilities for states A-E and death via chained standard gambles.
2. **Four societal inequality gambles**, again between triples of adjacent life states, to elicit utilities of states A-E and death via chained standard gambles.
3. **Four additional personal risk gambles** between randomly selected triples in which at least one state was non-adjacent, to allow testing the fit of the personal utility model through overidentification.

Personal risk gambles were presented first, as they were found in piloting to be more straightforward and hence familiarize participants with the task before presentation of the harder societal inequality gambles. The third block of non-adjacent gambles, deemed less central to the study, was positioned last to minimise the risk of survey fatigue during the core tasks.

Rather than ask participants for the exact probability at which they are indifferent to the gamble, we adopted a descending-probability procedure, in which gamble were first presented with a 1-in-2 chance of the worse outcome, followed by progressively lower probabilities: 1/5; 1/10; 1/100; 1/1,000; 1/10,000; 1/100,000; 1/1,000,000. If a participant refused a gamble, the next lower probability was shown. If they accepted a gamble (or refused the lowest possible odds) we proceeded to the next gamble. A ‘can’t choose’ option was available throughout. If this option was selected, the next lower probability was offered to check whether the respondent was at the indifference threshold. If indecision persisted, the gamble was marked as undecidable, and the next gamble was presented. Participants could revise previous responses at any point.

To further reduce cognitive load, the user interface presented information that remained unchanged between questions in ‘collapsed’ form. Details of previous life satisfaction ratings, or of the same gamble with a higher set of odds, were not displayed unless requested. Visual animations highlighted which elements had changed between questions (e.g. odds only, or both options and odds).

Consistent with FDA recommendations for risk communication (Food and Drug Administration (FDA), 2016), the gambles were presented with pictograms to illustrate the probability of each outcome. In line with the principle of eliciting informed preferences (Frijters & Krekel, 2021) we also provided real-world comparators for each probability. For example: “For comparison, a UK adult aged 20–49 has a 1 in 100 chance of dying over a 10-year period.”

Full examples of the gamble tasks are provided in the Supplementary Materials.

### Analysis of Standard Gambles

For Objective 2, we derive both utilities and a loss aversion measure from gamble responses. As described in Sect. 2.1, we take a theory-agnostic approach. This begins with analysis of the standard gamble under EUM based on Gafni (1994). If a participant *i* is indifferent to the choice of a fixed outcome, versus a gamble with probability $\:{p}_{i}$ of a worse outcome, then we can relate their personal utilities of the win, lose and baseline states ($\:{U}_{w,i},\:{U}_{l,i},\:{U}_{b,i}$) by Eq. 1:

1 $$\:{U}_{b,i}={p}_{i}{U}_{l,i}+\left(1-{p}_{i}\right){U}_{w,i}$$

For an ordered series of states, we define in ascending order for each participant $\:{U}_{Fi},\:{U}_{Ei},\:{U}_{Di},\:{U}_{Ci},{U}_{Bi},{U}_{Ai}$. In the current study $\:{U}_{F}$ represents the utility of death. The utility scale is arbitrary in location and scale, so for the purpose of reporting results we fix two values $\:{U}_{F}=0,{U}_{A}=1$. However for estimation, we fix $\:{U}_{F}=0,{U}_{E}=1$ both to ensure a well-conditioned optimization, and to avoid the definition of $\:{U}_{E},\:{U}_{D},\:{U}_{C},{U}_{B}$ as arbitrarily close to 1, where rounding errors can cause these utilities to appear equal in the presence of high levels of risk aversion. Utilities are rescaled between estimation and reporting. As we don’t directly elicit a point of indifference from participants, we rely on observed choice data giving the highest known probability of failure at which each participant accepts each gamble (taken to be zero if they reject *p*=10^− 6^), and the lowest at which they reject it (taken to equal one if they accept the first gamble *p*=1/2). The point of indifference is therefore taken to be the midpoint of these values on a log scale.

To incorporate probability weightings from CPT, we simplify the standard CPT approach. CPT specifies a reference point, from which losses and gains are usually weighted differently. In a standard gamble framing, the certain outline $\:{U}_{b,i}$ is usually assumed to be the reference point (Oliver, 2003). The equivalent relation is then expressed as Eq. 2:

2 $$\:0={w}_{i}^{-}\left({p}_{i}\right){v}_{i}^{-}\left({U}_{l,i}-{U}_{b,i}\right)+{w}_{i}^{+}\left(1-{p}_{i}\right){v}_{i}^{+}\left({U}_{w,i}-{U}_{b,i}\right)$$

where $\:{v}_{i}^{-},{v}_{i}^{+}$ are functions for each participant which allow for heterogenous valuation of losses and gains respectively, to capture loss aversion effects, while $\:{w}_{i}^{-},{w}_{i}^{+}$ allow weighting of loss and gain probabilities respectively, to capture their over- or under-weighting of small probabilities. In the current study, we make use of probability weighting functions $\:{w}_{i}^{-},{w}_{i}^{+}$ to test sensitivity of our results to small probability weighting effects. The loss and gain functions are, however, removed as (i) we are considering only point estimates of utility rather than a continuous function, and (ii) we seek a theory-agnostic common ground between EUM and CPT. We therefore take $\:{v}_{i}^{+}\left(x\right)={v}_{i}^{-}\left(x\right)=x$ and capture the relative valuation of losses compared to gains, $\:\lambda\:$, in a gamble between equal gains and losses - regardless of whether defined by CPT or EUM – by Eq. 3:

3 $$\:\lambda\:=\:\frac{{U}_{b,i}-{U}_{l,i}}{{U}_{w,i}-{U}_{b,i}}$$

Note therefore that while EUM accepts the computed $\:\lambda\:$ at face value, CPT may consider some component of the computed $\:\lambda\:$ as bias rather than ‘true’ preference. Noting also that $\:0<\lambda\:<\infty\:$ (with $\:\lambda\:=1$ representing risk neutrality) we apply a log scale to create a symmetric measure centred on zero, followed by a standard logistic function to ensure finite bounds. In combination, these operations lead to Eq. 4:

4 $$\:{\lambda\:}^{{\prime\:}}=\frac{\lambda\:-1}{\lambda\:+1}$$

a measure in which $\:-1<{\lambda\:}^{{\prime\:}}<1$, $\:\lambda\:=0$ represents risk neutrality, and $\:\lambda\:=\pm\:x$ represents equal degrees of risk aversion/risk seeking on each side of the scale. When summarizing risk aversion tendencies, we take the mean value of $\:{\lambda\:}^{{\prime\:}}$ over all relevant gambles.

The chained standard gamble gives only a point estimate for each item on a scale, with an exactly identified model lacking any quantification of error. We therefore also assess fit of the personal utility models by presenting further gambles, randomly selected from all gambles for states up to 3 steps apart on the ordinal scale, including gambles with asymmetric wins and losses. We fit the set of personal gambles for each participant using the binomial logit discrete choice model shown in Eq. 5:

5 $$\:P\left({C}_{i,gamble}\right)=\frac{\mathrm{e}\mathrm{x}\mathrm{p}\left({\sigma\:}_{i}{U}_{i,gamble}^{}\right)\:}{\mathrm{exp}\left({\sigma\:}_{i}{U}_{i,gamble}^{}\right)+\mathrm{e}\mathrm{x}\mathrm{p}\left({\sigma\:}_{i}{U}_{i,baseline}^{}\right)}$$

where $\:P\left({C}_{i,gamble}\right)$ is the probability of participant *i* choosing a given gamble with expected utility $\:{U}_{i,gamble\:}$ over a fixed outcome with utility $\:{U}_{i,baseline}$. $\:{\sigma\:}_{i}$ is a choice sensitivity parameter unique to the participant, estimated by maximum likelihood for each participant along with their personal utilities for states $\:{U}_{Di},\:{U}_{Ci},{U}_{Bi},{U}_{Ai}$ (fixing $\:{U}_{F}=0,{U}_{E}=1$ for estimation). Individual-level estimation is preferred over a random effects model, to fully visualise participant heterogeneity in utility curves; for this purpose we find the higher variance implicit in individual models to be acceptable.

For Objective 4 – the test of sensitivity to small probability weighting under CPT - although the form of $\:{w}_{i}^{-},{w}_{i}^{+}$ has high potential to affect results, we did not find any literature making empirical tests for values of these functions below *p* = 0.01. Noting that the current study uses probabilities as low as *p* = 10^− 6^, we can at best test sensitivity of our results to the extrapolated shape of $\:{w}_{i}^{-},{w}_{i}^{+}$ defined in the literature. We base this on Gonzalez and Wu’s (1999) test of probability weights at *p* = 0.01 for the linear in log odds function given in Eq. 6:

6 $$\:{w}_{i}^{+}\left(p\right)={w}_{i}^{-}\left(p\right)=\frac{{\delta\:}_{i}{p}^{{\gamma\:}_{i}}}{{\delta\:}_{i}{p}^{{\gamma\:}_{i}}+{\left(1-p\right)}^{{\gamma\:}_{i}}}$$

where $\:{\delta\:}_{i},{\gamma\:}_{i}$ are parameters unique to each participant; in analysis we test values for both Gonzalez and Wu’s median participant, and their participant with greatest overestimation of small probabilities.

Analysis for all objectives is conducted in the Python/Jupyter ecosystem (Granger & Pérez, 2021; Kluyver et al., 2016) using SciPy (Virtanen et al., 2020), Pandas (McKinney, 2010; Pandas development team, 2020), statsmodels (Seabold & Perktold, 2010) and Matplotlib (Hunter, 2007).

## Results

### Response Validation

To assess comprehension and validate responses to the task, a supervised pilot was conducted with 20 participants via video call. This group demonstrated a good understanding of the survey instrument. Compared to the main, unsupervised sample, they took longer to complete the task (29 ± 11 vs. 23 ± 10 min; U = 3708, *p* = 0.02) and had slightly higher rates of failed attention checks (0.2 ± 0.4 vs. 0.07 ± 0.3; U = 3364, *p* = 0.03). However, there were no significant differences in risk tolerance, model fit (McFadden’s r²), the proportion of choices correctly predicted by the standard gamble model, or task completion rates.

Three responses were removed from the main unsupervised sample due to dropped server connections, yielding a final sample size of *n* = 297. Among these, 8% completed all societal gambles but not all personal ones, 4% completed all personal gambles but not all societal ones, and 5% were unable to fully complete either set. Feedback from these participants cited a lack of contextual information, difficulty making decisions on behalf of others, or discomfort with mortality-related choices. When analysis was restricted to non-lethal gambles, completion rates increased to 90%. No significant differences in completion rates were observed by political affiliation.

**Fig. 2:**
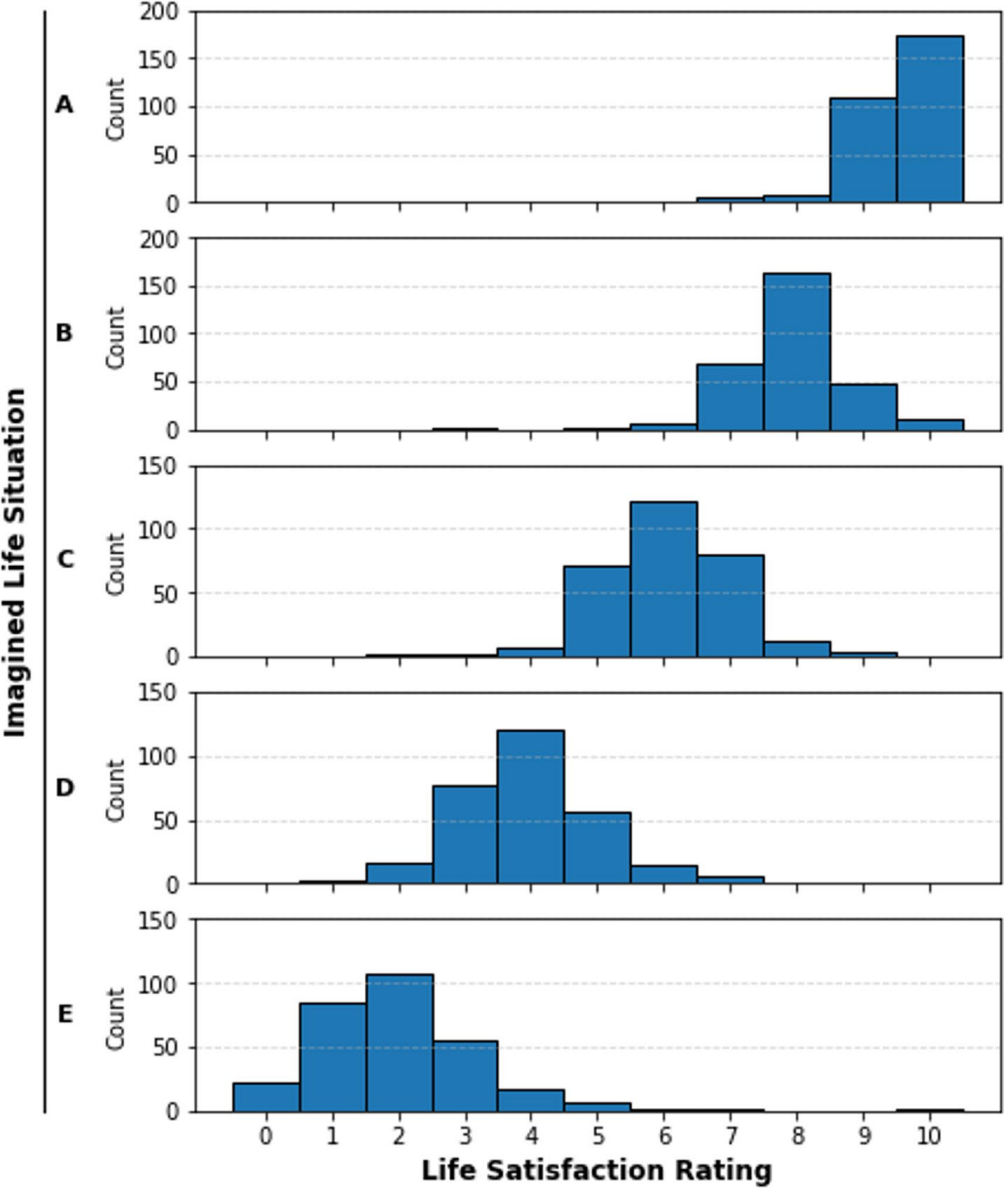
Distribution of life Satisfaction ratings for each imagined situation

The distribution of life satisfaction ratings given to each situation is shown in Fig. 2. Median life satisfaction ratings matched our own expectations for the imaginary scenarios, with A = 10, B = 8, C = 6, D = 4, E = 2.

Some participants (5.1%) rated some scenarios out of order. One of these participants admitted an error in their response but did not correct it; one claimed it matched their experience of people they know; and the remainder provided no explanation when prompted. During piloting, some participants had provided an explanation for such ratings, expressing a need to experience challenge rather than a “perfect life”. We discarded such responses as we consider them to represent a misunderstanding of what life satisfaction aims to measure. Results including these responses are shown in supplementary material, and are qualitatively similar.

No systematic differences were observed between the two survey conditions (life satisfaction before vs. after gambles) on any major outcome (completion time, risk tolerances, model fit).

Cronbach’s alpha was 0.70 for the five-item political scale, indicating acceptable internal reliability for a short scale.

### Estimation of Personal and Social Utilities, and Their Relationship with Political Alignment

**Fig. 3:**
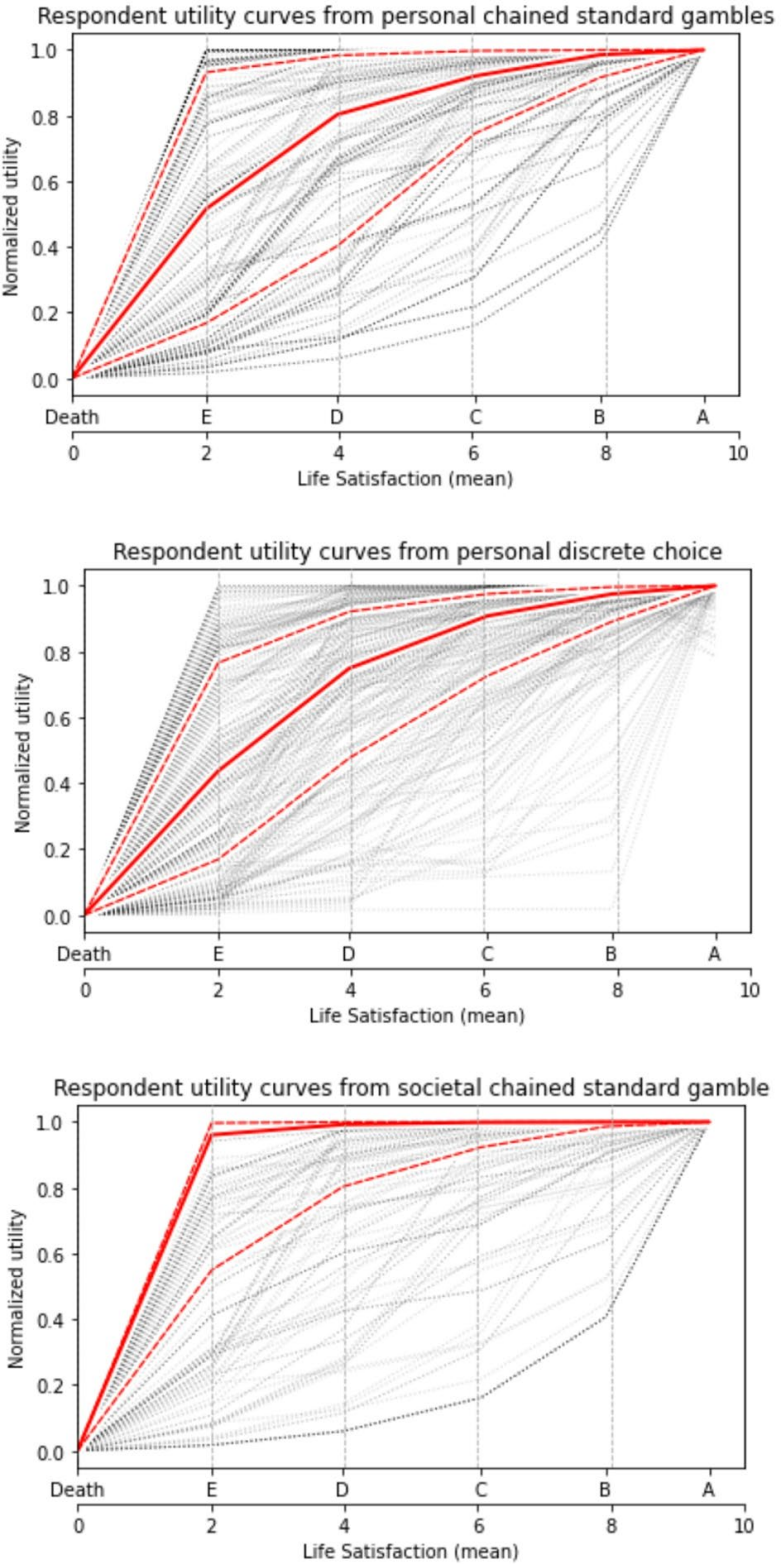
Individual participant utility curves derived under EUM. Red lines show median utility and quartiles. X positions are plotted at the mean life satisfaction rating for each scenario (A-E) over all participants. From top to bottom: (**1**) Utilities derived from 4 personal risk choices using chained standard gamble. (**2**) The same utilities derived from discrete choice modelling of 8 personal risk choices. (**3**) Utilities derived from 4 societal inequality choices using chained standard gamble

Estimated utilities of each situation are shown in Fig. 3, (i) for personal decisions fitted both by chained standard gamble and by discrete choice, (ii) for societal/policy maker decisions fitted by chained standard gamble. Goodness of fit for each personal discrete choice model is characterized by McFadden’s pseudo-r^2^. 53% of participants have an individual choice model with pseudo-r^2^ > 0.2, which is typically considered to represent good fit. The discrete choice model, fitted to 8 gambles, results in similar utilities to the chain of 4 gambles, though with greater smoothing and smaller variance between participants.

The societal utility curve is dominated by the high utility assigned to life itself, over any difference in life states. Some respondents in fact exhibit infinite risk aversion for societal gambles involving death. To capture their relative utilities for living states, it is therefore necessary to re-estimate utility curves excluding gambles with death.

We compute loss aversion ($\:\lambda\:$) for all gambles, with mean over gambles, and median over participants shown in Table 2. In all cases, the majority of participants demonstrate a clear aversion to both personal risk and societal inequality, with the latter being even more strongly avoided than the former. The aversion to societal inequality is most pronounced in gambles involving life-or-death outcomes (median $\:\lambda\:$=30.6) though still present in gambles between living states (median $\:\lambda\:$ for each gamble individually is 6.1). For personal choices, the median aversion to risk is lower for all states ($\:\lambda\:$=2.2).

**Table 2:** Median [Q1,Q3] (n) personal risk aversion (λ_p_) and societal inequality aversion (λ_s_) for all gambles between adjacent states. Percentage columns show proportion of participants exhibiting personal risk aversion, societal inequality aversion, and societal inequality aversion greater than personal risk aversion. Correlation columns use transformed values λ’_p_, λ’_s_ and show Pearson’s r (p-values) between personal risk aversion, societal inequality aversion and (leftwards) political alignment. Summary rows are derived from mean λ’ per participant, dropping participants who could not decide on all relevant gambles, then transformed back to λ for which we report the median over participants. All gambles (phys health) refers to gambles between physically healthy states A, B, C and D only

Gamble	λ_personal_ (*n*)	λ_societal_ (*n*)	λ_*p*_ > 1	λ_s_ > 1	λ_s_ ≥ λ_*p*_	*r*(λ’_*p*_,λ’_S_) (*p*)	*r*(λ’ _*p*_,politics) (*p*)	*r*(λ’ _S_,politics) (*p*)
E vs. D/Death	2.2 [0.4, 30.6] (273)	30.6 [2.2, 315.2] (269)	66%	84%	86%	0.46 (0.00*)	−0.03 (0.63)	−0.03 (0.61)
D vs. C/E	2.2 [0.4, 3.5] (278)	6.1 [2.2, 30.6] (276)	68%	81%	81%	0.50 (0.00*)	0.01 (0.84)	−0.04 (0.52)
C vs. B/D	2.2 [2.2, 6.1] (272)	6.1 [2.2, 30.6] (276)	74%	83%	81%	0.47 (0.00*)	−0.07 (0.27)	−0.08 (0.20)
B vs. A/C	2.2 [2.2, 30.6] (280)	6.1 [2.2, 315.2] (279)	79%	84%	79%	0.46 (0.00*)	−0.09 (0.12)	0.02 (0.79)
All gambles (phys health)	2.2 [1.0, 5.3] (271)	4.7 [1.8, 30.6] (275)	68%	83%	76%	0.58 (0.00*)	−0.09 (0.13)	−0.04 (0.50)
All gambles (no death)	2.2 [1.2, 4.0] (269)	4.0 [1.8, 13.7] (272)	79%	87%	73%	0.62 (0.00*)	−0.07 (0.25)	−0.05 (0.44)
All gambles	1.9 [1.1, 4.0] (263)	4.4 [2.0, 15.9] (261)	74%	82%	74%	0.63 (0.00*)	−0.06 (0.29)	−0.04 (0.50)

**Fig. 4:**
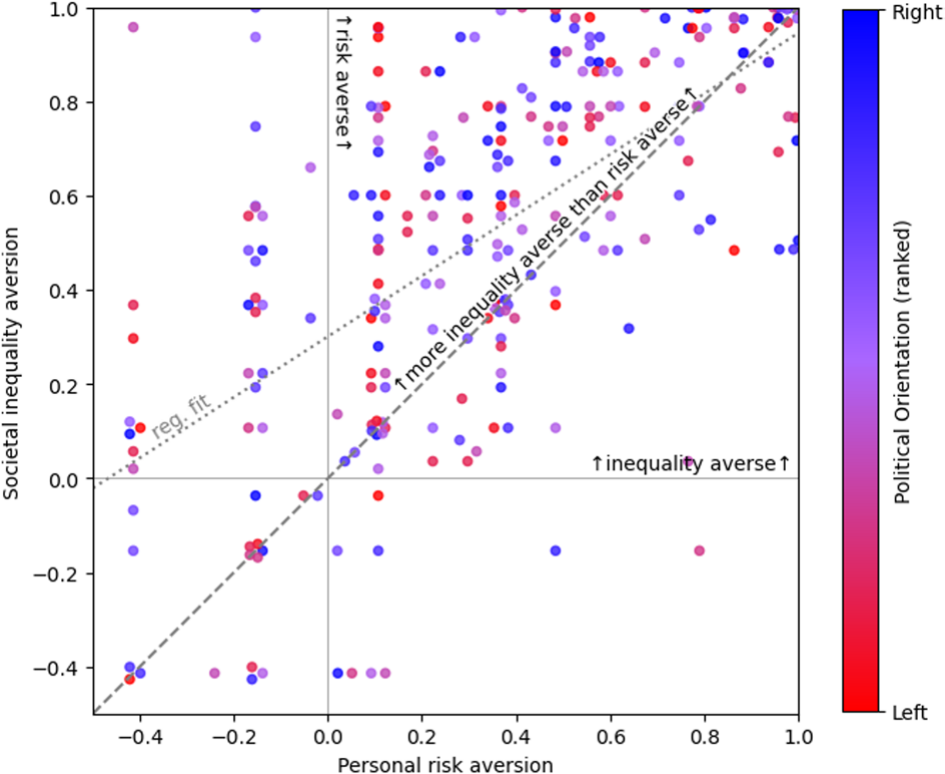
Scatterplot of personal risk aversion versus societal inequality aversion, estimated from chained standard gamble choices (excluding those with risk of death) and coloured by political alignment. The diagonal line indicates a position consistent with the veil of uncertainty. Dotted line shows regression fit. 41 sets of coincident points (108/271 points in total) have been displaced by 0.015 units for display

A plot of personal risk aversion versus social inequality aversion, for all gambles excluding death, is shown in Fig. 4, along with the political alignment of each participant. The principal findings are clearly visible: 79% of participants showed aversion to personal risk, while 87% showed aversion to societal inequality.

Although a veil-of-uncertainty philosophy would predict equal aversion to societal inequality and personal risk, 73% showed an aversion to societal inequality which is greater than their own aversion to personal risk. The two variables are nonetheless clearly correlated, with people more willing to take risks themselves, also more likely to take risks for others (*r* = 0.62, *p* < 0.01).

These tendencies are independent of political alignment, in particular, the correlation of societal inequality aversion and the political alignment scale is not significant (*r*=−0.05, *p* = 0.44). The Mann-Whitney U-test, applied to voters for each political party, also shows no significant relationship with societal inequality aversion, and only one significant result for personal risk aversion in a small sub-group (voting ‘Other’: *n* = 4, mean 0.72 ± 0.25 versus everyone else, *n* = 268, mean = 0.33 ± 0.34; U-statistic 858, *p* = 0.02).

To assist in interpreting these null results, we compute minimum detectable effects (MDE) that would in principle be significant given our sample, at *p* = 0.05. For the correlation analysis, the MDE would be a weak *r* = 0.12 overall. Although our correlation results are not significant, the coefficients found also run counter to the expected direction based on political narrative (right wing positions being more inequality averse) which further suggests it is unlikely that the null result is attributable to the study being under-powered. For individual party alignments, we compute MDE in median λ for the 3 largest parties (Conservative, Labour, Reform) to be at most 0.13. This compares to standard deviations of 0.34 (λ_personal_) and 0.37 (λ_societal_), i.e. within-group differences greatly exceed the minimum detectable between-group difference. For small parties, this test is inevitably less sensitive, and should not be overinterpreted for the smallest groups (our sample includes some responses for party vote with *n* = 1) however by definition these represent only a minority of respondents. Results and MDEs are similar if a t-test is used, but we report u-test results to avoid reliance on normality assumptions.

### Application of Estimated Utilities to National Life Satisfaction Data

To illustrate the implications of these results, explore the use of our estimated utilities to summarize the distribution of life satisfaction in the UK (Office for National Statistics, 2023). The mean of the current life satisfaction distribution is 7.45, which as we argued in the introduction, is not representative of the variance due to the nonlinear utility of life satisfaction. We therefore compute Representative Life Satisfaction (RLS) levels – constant values which, if experienced by everyone, are equivalent to the distribution observed by the ONS based on (a) personal risk choices and (b) societal inequality choices. Equivalence is defined in two ways: (i) using median utility curves, giving the lowest RLS that 50% of participants would consider better than the distribution; (ii) using mean utility curves, giving the same total utility as the distribution, and thus taking account of strength of preference for each participant.

To allow interpersonal comparisons, individual utility curves are normalized by standard deviation using the current UK distribution of life satisfaction as the reference distribution. Under a democratic interpretation, this gives each individual utility curve equal “voting” power (Cotton-Barratt et al., 2020). As the computation is sensitive to low utility states, for which ONS notes greater uncertainty in their estimates, the data has been binned to match ONS seasonally adjusted releases of life satisfaction data (Office for National Statistics, 2025) which defines ranges of 0–4, 5–6, 7–8, 9–10. As the ONS table covers only living states, we use the utilities we computed excluding gambles with risk of death.

Results are shown in Table 3. RLS varies depending on the measure used, but for all four cases, is lower than the distribution mean.

**Table 3:** Representative life satisfaction measures for the UK distribution of life satisfaction, 2023

Utility curve	Representative RLS (difference from mean LS of 7.45)
Personal risk, mean	6.99 (−0.46)
Personal risk, median	6.80 (−0.65)
Societal inequality, mean	6.53 (−0.92)
Societal inequality, median	5.69 (−1.76)

### Sensitivity to Small Probability Weighting

Given that CPT predicts participants may over-weight small probabilities in their decision-making, we provide two analyses of results from this perspective.

Firstly, we test sensitivity of the representative life satisfaction measures in Table 3, to application of probability weighting functions from CPT. Based on Gonzalez and Wu’s (1999) linear in log odds function, with median parameter values across their participants (δ = 0.77, γ = 0.44), we would expect w(10^− 6^) = 0.002, overweighting these odds by a factor of 2,000. Applying this probability weighting in the above analysis, we find a recomputed version of Fig. 4 to be qualitatively similar, albeit with all data points shrunk towards the origin. The reductions in RLS shown in parentheses in Table 3 reduce to the range [−0.30, −0.74]. Based on Gonzalez and Wu’s single participant with the most extreme parameters (δ = 1.19, γ = 0.27), we compute.

w(10^− 6^) = 0.03, overweighting these odds by a factor of 30,000. The recomputed Fig. 4 is still qualitatively similar, albeit with further shrinkage towards the origin, and the reductions shown in parenthesis in Table 3 shrink to at most −0.36. We remind the reader in interpretation that this is a sensitivity test based on extrapolated figures from the cited study only, as probability weightings for *p* < 0.01 have not been empirically measured in the literature.

Secondly, we compute residuals from both EUM and CPT models, disaggregated by probability of loss. All models slightly overestimated acceptance of very-low-probability risks; however, this pattern was limited to a small subset of trials (82% of test points had probability of loss ≥ 0.01). Importantly, CPT models, although explicitly accounting for small-probability distortion, did not improve overall model fit relative to EUM. Details are given in supplementary material.

## Discussion

Our findings show a concave (risk-averse) valuation curve of well-being states, and therefore qualitatively align with both Peasgood (2018) and Mukuria (2023). However, we observe a higher degree of personal risk aversion than either study. This may be partly due to the overweighting of small probabilities; when this is accounted for in a sensitivity analysis, our estimates of personal risk aversion align more closely with those reported by Mukuria (excluding states worse than dead, which fall outside the scope of our study; note also that this comparison relies on an untested assumption of the EQ-HWB-S scale having a linear relationship with Life Satisfaction). In contrast, Peasgood reports milder risk aversion, potentially due to the lack of objective anchors for scale points, a feature incorporated in both Mukuria’s study and our own. Notably, neither Peasgood nor Mukuria examined decision-making in interpersonal contexts. In such settings, our results indicate substantially greater societal risk aversion, suggesting individuals may adopt more cautious preferences when outcomes affect others rather than themselves.

Considering the representative life satisfaction measures computed, the largest RLS reduction can be interpreted as the average loss in life satisfaction that a typical participant might accept as a trade-off for achieving population-level equality in well-being - a striking figure of 1.8 points on the scale of 10. However, the preference difference between a 1.8-point and a 0.9-point life satisfaction reduction is marginal for the median participant. Under a democratic interpretation, where utility curves are normalized so that each individual curve has equal “voting” power (Cotton-Barratt et al., 2020), most of the median “vote” is used to express strong preference for avoiding the lowest life satisfaction states. By definition this means expressing relative indifference between higher states, and therefore a relatively high utility for the current state of the UK, where 94% of the population report a life satisfaction of 5 or higher. The mean utility approach captures not only the direction but also the intensity of individual preferences, resulting in less pronounced differences between mean life satisfaction and RLS. Even so, RLS based on mean utility still implies average willingness to trade away 0.9 points of personal life satisfaction in exchange for well-being equality, which is substantial.

Political orientation has long been known to be influenced by cultural and social factors, and for many voters is more a question of identity than policy preference (Converse, 2006). Nonetheless it is surprising to see no correlation whatsoever of social inequality aversion with politics. We suggest two possible reasons for this: firstly, that the posing of these questions in the life satisfaction domain – rather than the financial domain – may serve to decouple respondents’ views from entrenched economic ideologies, partisan cues, and known financial reference points such as the participant’s own income, or the minimum wage (Fazio & Reggiani, 2023). Secondly, the fact that well-being outcomes are framed *without reference to its current social distribution*, removes any reference point that would allow the participant to use their own political opinions on the status quo as a proxy for their judgement of our scenarios, and thus helps to uncover preferences on an absolute scale.

The existing consensus is that redistribution preferences are linked not only to political ideology (Alesina & Giuliano, 2011), but also other factors such as intergenerational mobility (Alesina et al., 2018; Shariff et al., 2016) or perception of the underlying causes of inequality (Cappelen et al., 2010). It should be noted that while this literature focuses on redistribution preference - a question posed at the policy level - we focus on preference regarding inequalities in life satisfaction, a measure targeted at the outcome level. As such, our findings are orthogonal to those relating to the policy question of how desired outcomes should be obtained. By showing independence from political alignment at the outcomes level, however, this result does support an interpretation of the existing literature in which political ideology is itself more strongly linked to beliefs on what constitutes effective policy, than it is linked to differences in outcome preferences (Johnston et al., 2025).

More generally, existing research shows that national inequality tends to be negatively correlated with individual well-being (Buttrick et al., 2017; Ugur, 2021). Our finding of inequality-averse utility curves broadly corroborates such research. By framing the problem as an efficiency/equity trade-off, we extend previous findings by quantifying a theoretical limit beyond which further reductions in inequality would not justify further sacrifices in average life satisfaction; notwithstanding, we also establish from analysis of RLS that the UK could still substantially reduce life satisfaction inequality before reaching such a limit.

## Strengths & Limitations

We have proposed an RLS measure which differs from more conventional approaches that apply standard inequality measures such as the Gini coefficient, variance, or the Atkinson index. These traditional measures implicitly reflect assumptions about how much inequality matters; RLS, by way of contrast, is based on directly measuring how much inequality matters through modelling individual decisions. Compared to averages such as mean life satisfaction or WELLBY-style measures, RLS explicitly takes the distribution of wellbeing into account, without requiring the researcher to choose an arbitrary inequality aversion parameter. Nonetheless it is presented as a complement to, rather than replacement for existing metrics. We note that RLS is quite sensitive to small changes in the distribution due to a double source of nonlinearity: (i) the transformation from life satisfaction to utility, and (ii) the transformation from utility back to life satisfaction, using a different part of the same utility curve. When comparing multiple different policy outcomes, therefore, an average of normalized utility values is likely to be a better conditioned metric compared to RLS.

In a wider context, this evidence is highly relevant to the political questions of how governments should weigh alternative courses of action. While a full exploration of this question is clearly beyond our scope, its relevance warrants a reflection on the limitations of interpreting our findings not only from an empirical, but also a normative perspective.

Taking a policy perspective, most participants exhibit risk aversion. Interpreting this tendency depends on a normative stance: whether such aversion reflects a cognitive bias in line with normative application of Cumulative Prospect Theory (CPT), or a legitimate expression of collective preference, consistent with Expected Utility Maximization (EUM). How policymakers treat this aversion (as a bias, or a normatively valid preference) will drastically affect their recommendations.

In the context of political application, it should be noted that self-reported wellbeing measures are susceptible to bias from (i) the expectations individuals have for their lives, (ii) the incentive to misrepresent ones own well-being in order to influence policy (we note that within the current study, this latter limitation may have biased the reporting of ‘zero’ life satisfaction in the UK ONS data). Any attempt to use well-being measures in policymaking must therefore take account of these factors. Where unadjusted well-being measures are used, this relies on the reasonableness of people’s life expectations being constant across all demographics. Aggregate approaches based on data collected outside of the political context are likely to be more reliable.

The current study focuses on outcomes and not process (ends and not means): in reality both are important. Future models could consider dynamic changes in well-being over time rather than a static endpoint, and also the effect of repeated gambles. This would also provide a framework for incorporating the effect of expectations on well-being.

The study is based on a UK sample and would benefit from internationalization; traditional probability sampling would also enhance the representativeness albeit at greater cost. Political orientation is a complex construct (Feldman & Johnston, 2014) and replication efforts would benefit from more comprehensive measurement strategies, which will be particularly crucial in international contexts. The survey would benefit from presenting participants with more gambles on societal inequality, allowing for the fit of a discrete choice model to predict societal inequality tolerance as well as personal risk tolerance. Our use of a medical framing for personal risk decisions, although justified in terms of response efficiency, represents a limitation, as responses may vary across domains. Future work could test the robustness of these findings to alternative framing. Further nuance could also be obtained by studying the effect of removing the veil of uncertainty, i.e., using a framing in which participants are told they will not be personally affected by a policy choice. However, given that we found most participants to be more sensitive to social inequality than to personal risk, we would not expect this particular change to elicit substantially different results. Although our core conclusions remain robust across all models tested, further research is recommended to experimentally test the degree to which participants over-weight the very small probabilities used in this study, and to explore alternative preference elicitation methods such as multi-component choices and time trade-off, to narrow the range of estimates for utility curves.

We did not present participants with life states considered worse than death. Although Peasgood et al. (2018)’s participants rate death as equivalent to approximately 2/10 on the life satisfaction scale, our own participants report high aversion to mortality risk even from a baseline of 2/10. More work is therefore needed to determine risk aversions between different low-satisfaction states, and between low-satisfaction states and death.

The reliance on stated preference is an expected limitation. From an empirical, behaviourist perspective, future research could augment this with a revealed preference approach. However from a policy perspective, this same limitation is inherent in the democratic process itself. It may therefore be fruitful to compare existing consensus decisions made by the public, to the optimal-utility decisions predicted under this framework.

As with any psychological study, it is important to consider whether some of the observed effects may reflect artefacts of the task design rather than genuine underlying preferences. In particular, although our participants are themselves anonymous, their task is to imagine themselves as a policymaker, whose decisions - participants may plausibly assume - might be known to the imagined public. Participants are thus asked to imagine themselves in a situation which evokes imagined social desirability bias. To mitigate this effect, we (i) present personal risk and societal inequality results side-by-side, (ii) report analyses excluding death-risk gambles, and (iii) emphasise the need for future work using group-based or consensus-building designs that may better isolate distributive preferences from responsibility-aversion. Following best practice in the field, independent replication of these results in different contexts would nonetheless be of value.

## Conclusions

This study takes an empirical measurement of how people say they would balance risk, reward, equality and efficiency in well-being trade-offs concerning both themselves and others. From a political perspective, these findings are noteworthy. The traditional left-right political spectrum is often seen as capturing the degree to which individuals are willing to trade total wealth for more equal outcomes. The current study measures an analogous trade-off in the domain of well-being. We find that 87% of participants choose to make this trade-off to some extent, and, importantly, that the degree of trade-off each person prefers is unrelated to their political alignment.

In a time of sharp ideological divides over inequality, this suggests a unifying human intuition that transcends political ideology. Rather than attempting to reconcile polarised policy demands, decision-makers might instead build on shared intuitions about fairness in well-being as a basis for consensus.

With regard to metrics, the study shows that mean life satisfaction is not an adequate summary of well-being distribution. Representative Life Satisfaction (RLS) scores were found to be up to 1.8 scale points lower. We therefore propose the adoption of an RLS-based metric for national well-being reporting, and suggest adjustments to the WELLBY measure to account for the nonlinear utility effects observed in our data.

The question naturally arises as to which RLS measure to use. Although we have made progress in defining these measures based on public preference, we consider the final choice to require further research. Economists wishing to integrate our findings with a classical maximisation of utility will likely prefer a measure based on mean participant utilities, though an ethical question remains over whether they should be computed from personal or societal gambles, and a further consideration is the choice of reference distribution when normalizing utilities. Measures based on the median respondent bypass the issue of the reference distribution, but ignore strength of preferences.

Taking a broader view, the question of mean versus median RLS is a narrow attempt to answer the wider question of how we should make decisions when individuals with different utility curves disagree. Given the wide range of risk tolerances exhibited by our participants, a fruitful avenue for future research will be to explore how groups can reach consensus decisions under such divergent preferences. On this topic, we can ask both quantitative questions (at what odds is each participant prepared to compromise?) and qualitative ones: what do people perceive as the ethical issues surrounding such a consensus building process? Why are we more careful with the lives of others than with their own? Such care may simply reflect a precautionary principle learned in real conditions, where outcomes are less certain than in a stated preference experiment. Alternatively, it may reflect either social desirability bias, or uncertainty in the preferences of others – in which case the differences between personal risk aversion, and societal inequality aversion, may disappear in the context of a group consensus-building exercise.

There are many ethical decisions which people think are wrong to make on the basis of utilitarian, numerical assessment of outcomes, whether or not adjusted for risk or inequality. Our findings do not change that. Human rights are considered to be inalienable, regardless of any utility calculation. The remit of a democratic government is limited by design, and overreach of powers is considered wrong, regardless of any utility calculation. Further examples exist where utility calculations are widely considered to be inappropriate.

Notwithstanding, there are many areas where decision-making by numbers is already widely accepted. Cost-benefit analysis, for instance, is generally seen as a legitimate framework, even if the details of specific calculations are subject to debate. Health economics - particularly in its valuation of human life - may attract more public controversy, yet it remains a well-established contributor to policymaking. In domains such as transport, computational simulations have long been used to support planning decisions rather than replace planners. In these, and similar areas, the present study offers both a methodological framework, and empirical grounding, to help ensure that the values embedded in numerical calculations better reflect the preferences of the wider public.

## Supplementary Information

Below is the link to the electronic supplementary material.

Supplementary file 1 (DOCX 174 KB)

## Data Availability

All data supporting this study is openly available in OSF data repository at 10.17605/OSF.IO/UZFY7
