# Supplementary material for: Individual Utilities of Life Satisfaction Reveal Inequality Aversion Unrelated to Political Alignment
Source: Soc Indic Res. 2026 May 13;183(1):12. doi: 10.1007/s11205-026-03854-4 (PMC13172002; doi:10.1007/s11205-026-03854-4)
Supplement: Supplementary file 1 — (DOCX 174 KB) [file 11205_2026_3854_MOESM1_ESM.docx]

# Individual utilities of life satisfaction reveal inequality aversion unrelated to political alignment: Supplementary material

## Sample survey questions

### Personal risk gamble, life satisfaction basis

Now, consider the following scenario.

Imagine you are in a situation where you rate your life satisfaction as 8 out of 10.

Imagine that all your life, you have had a chronic health condition which you were born with, and which restricts your life somewhat. One day your doctor says you must choose between two treatments for this condition.

- Treatment A has a guaranteed outcome. You have thought about this outcome and you are certain that your life satisfaction after treatment would be 8 (out of 10).
- Treatment B is potentially better, but carries risk - the evidence shows that for some people it fails and makes the condition worse. You think that if the treatment succeeds, your life satisfaction would improve to 10, but if it fails, your life satisfaction would deteriorate to 6.


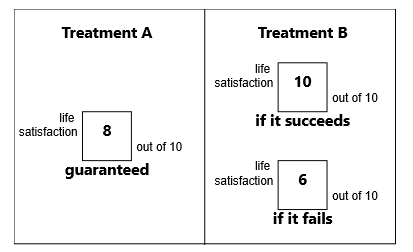


(*If you need to see how you rated different people's life satisfaction again, click* HERE)


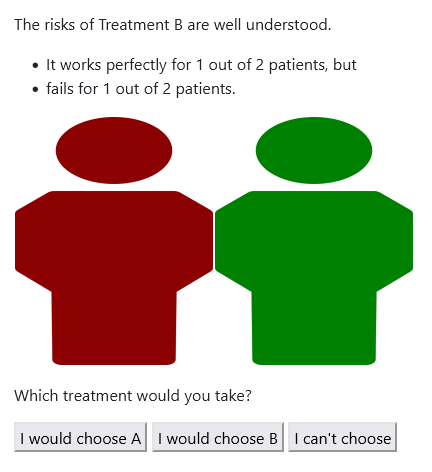


### Policymaker scenario – life satisfaction basis

Now, consider the following **new** scenario.

**The context of this scenario is different - please read carefully!**

Imagine you are a **policymaker** who must make a choice affecting a large number of people. Currently, everyone in the affected group rates their life satisfaction as 2.

You have the following options:

- Policy A has a guaranteed outcome. Research shows that everyone's life satisfaction afterwards would be 2 (out of 10).
- The outcome of Policy B varies between people. Many people will benefit, and their life satisfaction afterwards would be 4 (out of 10). Others, however, would die as a result of the new policy. You cannot tell beforehand who would live or die.


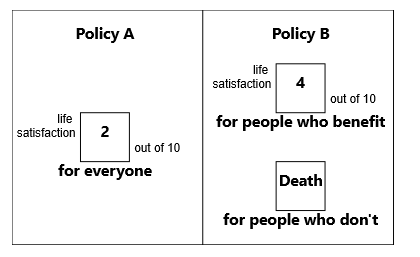


*You said you would choose Policy A.*

However, suppose the effects of Policy B were better, and it would

- benefit **99** out of **100** people, but
- the people it does not benefit (**1** out of **100** people) will die.

*The policy will affect you as well, though you don't know yet whether you will benefit or die.*


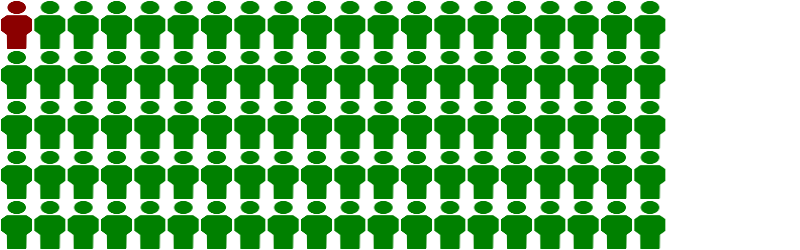

*For comparison, in everyday life, a UK adult (age 20-49) has a 1 in 100 risk of dying every 10 years.*

Which policy would you choose now?


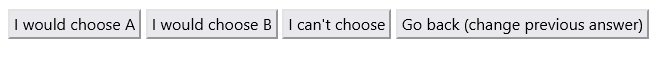


### Personal risk scenario – vignette basis

Now, consider this scenario, based on the following life situations:

| **Situation** | **Career** | **Relationships** | **Physical Fitness** |
| --- | --- | --- | --- |
| D | I don't enjoy my job. The pay is low, and sometimes I struggle to pay the bills. | I only occasionally visit my family and friends, and I often feel lonely. I am unhappy about my relationship status. | I rarely exercise. I would like to exercise more but I struggle to find time and motivation. |
| E | I would like to work, but I am physically unable to. The state provides some benefits to help cover essentials, but I often struggle to pay the bills. | I am unable to visit family or friends, and I always feel lonely. I am very unhappy about my relationship status. | I would like to play sports but I am not physically able to do so. |

Imagine yourself living a life similar in overall quality to **Situation E**. *Your own life priorities may differ from Career, Relationships & Fitness, but please imagine yourself succeeding or struggling in areas that matter most to you, to a similar extent.*

Imagine that all your life, you have had a chronic health condition which you were born with, and which restricts your life somewhat. One day your doctor says you must choose between two treatments for this condition.

- Treatment 1 has a guaranteed outcome. Your life would remain comparable to **Situation E**.
- Treatment 2 has greater potential for improving your condition, although carries a risk of death. However if it succeeds, you think your life would improve to something like **Situation D**.


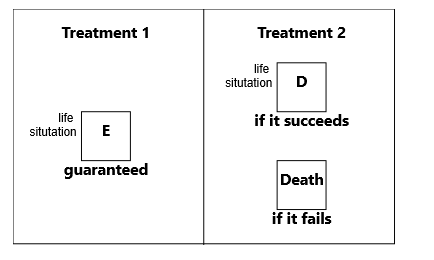


### Political alignment

The political questions are as follows.

- Regarding politics, do you consider yourself more left leaning or right leaning?
- For each of the following statements, please select your level of agreement:
  - Government should redistribute income from the better off to those who are less well off
  - Big business benefits owners at the expense of workers
  - Ordinary working people do not get their fair share of the nation’s wealth
  - There is one law for the rich and one for the poor
  - Management will always try to get the better of employees if it gets the chance

## Analysis of residual split by probability of loss

Figure S1 shows a visualisation of model residuals split by probability of losing the presented gamble. Consistent with the predictions of CPT, a trend is visible in which all models (both EUM and CPT) overestimate the tendency of participants to accept gambles with small loss probabilities. Although the CPT models used do reduce this tendency, they do not fit the data better overall: mean square error for EUM was 0.161, and for the two CPT models presented 0.170 and 0.177 respectively (where mean square error of 1 corresponds with complete misprediction). The larger residuals represent a minority of gambles tested (18% have loss probability <0.01).


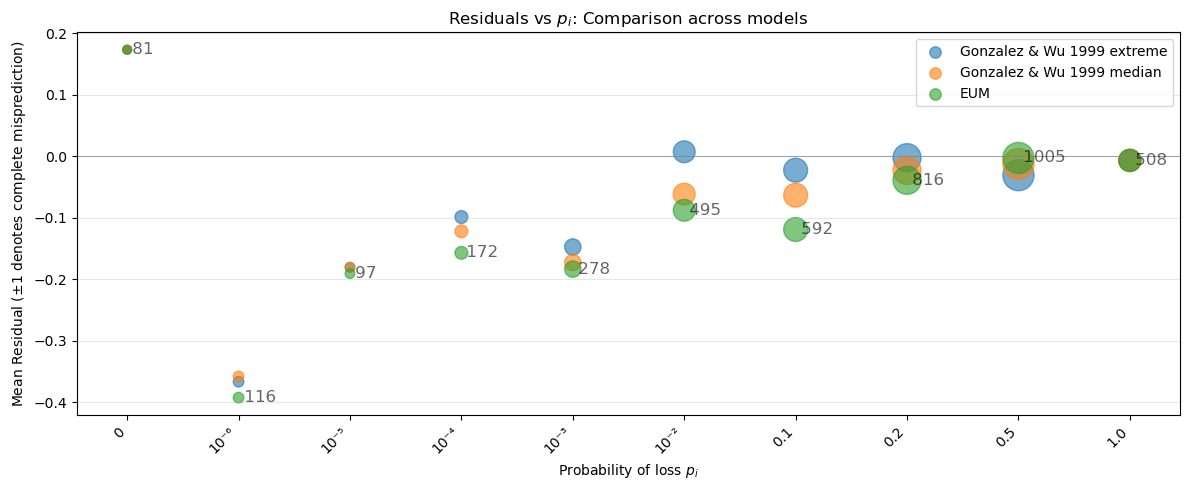


**Fig. S1** Mean residuals of choice models split by probability of loss. Marker size and labels show the number of data points in each case. EUM denotes Expected Utility Maximization model; CPT models are taken from (i) the median and (ii) the most extreme participant in Gonzalez & Wu 1999.

## Main results including responses with out-of-order life satisfaction ratings

Table S1 shows the equivalent to Table 2, albeit with responses included where participants did not rate improved life situations as having increasing life satisfaction. Note that this is only possible for the gambles-first survey condition, as in the life satisfaction-first survey condition we routed such participants directly to demographic questions (skipping gamble questions). The number of participants is thus approximately half of that shown in Table 2. The findings remain qualitatively similar.

| Gamble | λ_personal_ (n) | λ_societal_ (n) | λ_p_>1 | λ_s_>1 | λ_s_≥λ_p_ | r(λ'_P_,λ'_S_) (p) | r(λ' _P_,politics) (p) | r(λ' _S_,politics) (p) |
| --- | --- | --- | --- | --- | --- | --- | --- | --- |
| E vs D/Death | 2.2 [0.4, 30.6] (150) | 30.6 [2.2, 315.2] (153) | 65% | 82% | 84% | 0.47 (0.00*) | -0.06 (0.43) | -0.06 (0.44) |
| D vs C/E | 2.2 [0.4, 2.2] (154) | 6.1 [2.2, 30.6] (155) | 72% | 83% | 81% | 0.43 (0.00*) | -0.01 (0.95) | -0.08 (0.30) |
| C vs B/D | 2.2 [2.2, 6.1] (153) | 6.1 [2.2, 30.6] (152) | 76% | 82% | 79% | 0.42 (0.00*) | -0.08 (0.31) | -0.07 (0.41) |
| B vs A/C | 2.2 [2.2, 30.6] (156) | 6.1 [2.2, 30.6] (154) | 79% | 83% | 76% | 0.39 (0.00*) | -0.11 (0.18) | 0.06 (0.49) |
| All gambles (phys health) | 2.2 [1.0, 6.1] (153) | 3.4 [1.7, 30.6] (151) | 70% | 82% | 74% | 0.55 (0.00*) | -0.11 (0.18) | -0.01 (0.86) |
| All gambles (no death) | 2.2 [1.2, 4.0] (151) | 3.5 [1.7, 13.7] (151) | 80% | 86% | 73% | 0.58 (0.00*) | -0.10 (0.24) | -0.05 (0.58) |
| All gambles | 1.9 [1.1, 4.6] (146) | 4.0 [1.8, 12.8] (149) | 73% | 83% | 72% | 0.62 (0.00*) | -0.10 (0.21) | -0.06 (0.47) |

Table S1 For all respondents (including those with out-of-order life satisfaction ratings), median [Q1,Q3] (n) personal risk aversion (λp) and societal inequality aversion (λs) for all gambles between adjacent states. Percentage columns show proportion of participants exhibiting personal risk aversion, societal inequality aversion, and societal inequality aversion greater than personal risk aversion. Correlation columns use transformed values λ'p, λ's and show Pearson’s r (p-values) between personal risk aversion, societal inequality aversion and (leftwards) political alignment. Summary rows are derived from mean λ' per participant, dropping participants who could not decide on all relevant gambles, then transformed back to λ for which we report the median over participants. All gambles (phys health) refers to gambles between physically healthy states A, B, C and D only.
